# Expression and methylation data from SLE patient and healthy control blood samples subdivided with respect to ARID3a levels

**DOI:** 10.1016/j.dib.2016.08.049

**Published:** 2016-08-31

**Authors:** Julie M. Ward, Michelle L. Ratliff, Mikhail G. Dozmorov, Graham Wiley, Joel M. Guthridge, Patrick M. Gaffney, Judith A. James, Carol F. Webb

**Affiliations:** a Department of Medicine, Oklahoma University Health Sciences Center, Oklahoma City, Oklahoma, USA; b Department of Biostatistics, Virginia Commonwealth University, Richmond, Virginia, USA; c Arthritis and Clinical Immunology Research Program, Oklahoma Medical Research Foundation, Oklahoma City, Oklahoma, USA; d Department of Pathology, Oklahoma University Health Sciences Center, Oklahoma City, Oklahoma, USA; e Department of Microbiology and Immunology, Oklahoma Health Sciences Center, Oklahoma City, Oklahoma, USA; f Department of Cell Biology, Oklahoma University Health Sciences Center, Oklahoma City, Oklahoma, USA

**Keywords:** SLE, B cells, ARID3a

## Abstract

Previously published studies revealed that variation in expression of the DNA-binding protein ARID3a in B lymphocytes from patients with systemic lupus erythematosus (SLE) correlated with levels of disease activity (“Disease activity in systemic lupus erythematosus correlates with expression of the transcription factor AT-rich-interactive domain 3A” (J.M. Ward, K. Rose, C. Montgomery, I. Adrianto, J.A. James, J.T. Merrill et al., 2014) [1]). The data presented here compare DNA methylation patterns from SLE peripheral blood mononuclear cells obtained from samples with high numbers of ARID3a expressing B cells (ARID3a^H^) versus SLE samples with normal numbers of ARID3a^+^ B cells (ARID3a^N^). The methylation data is available at the gene expression omnibus (GEO) repository, “Gene Expression Omnibus: NCBI gene expression and hybridization array data repository” (R. Edgar, M. Domrachev, A.E. Lash, 2002) [2]. Isolated B cells from SLE ARID3a^H^ and ARID3a^N^ B samples were also evaluated via qRT-PCR for Type I interferon (IFN) signature and pathway gene expression levels by qRT-PCR. Similarly, healthy control B cells and B cells stimulated to express ARID3a with the TLR agonist, CpG, were also compared via qRT-PCR. Primers designed to detect 6 IFNa subtype mRNAs were tested in 4 IFNa, Epstein-Barr Virus-transformed B cell lines (“Reduced interferon-alpha production by Epstein-Barr virus transformed B-lymphoblastoid cell lines and lectin-stimulated lymphocytes in congenital dyserythropoietic anemia type I” (S.H. Wickramasinghe, R. Hasan, J. Smythe, 1997) [3]). The data in this article support the publication, “Human effector B lymphocytes express ARID3a and secrete interferon alpha” (J.M. Ward, M.L. Ratliff, M.G. Dozmorov, G. Wiley, J.M. Guthridge, P.M. Gaffney, J.A. James, C.F. Webb, 2016) [4].

**Specifications Table**

**Table:** 

Subject area	*Immunology*
More specific subject area	*SLE and ARID3a*^*+*^*B cells*
Type of data	*Figure, Tables, link*
How data was acquired	*Electrophoresis and BIOMARK HD*
Data format	*Raw, analyzed*
Experimental factors	*FACS-purified SLE and healthy B lymphocytes (+/- CpG-stimulation)*
Experimental features	*DNA was isolated from ARID3a*^*H*^*and ARID3a*^*N*^*total PBMCs; RNA was extracted from LCLs, peripheral blood SLE B cells, and healthy control B cells with or without CpG-stimulation for 24 hours.*
Data source location	*Oklahoma City, OK; USA*
Data accessibility	*Data is available within this article and deposited in NCBI׳s Gene Expression Omnibus*http://www.ncbi.nlm.nih.gov/geo/ accessible*via GEO series accession number: GEO:*GSE84965

**Value of the data**

• DNA gene methylation data derived from SLE peripheral blood mononuclear cells were subdivided based on levels of ARID3a expression, a transcription factor which correlated with disease activity indices [1], allowing comparison of patient samples with high and low ARID3a levels.
• Data for expression of a subset of IFNa associated genes obtained from SLE samples with high or low ARID3a expression, and from healthy control blood cells or those stimulated to express increased levels of ARID3a, allow comparison of effects of high and low ARID3a expression on gene expression.
• Data provide validation of primer sets useful for studying Type I interferon signature genes.

## 1 Data

**Fig. 1:**
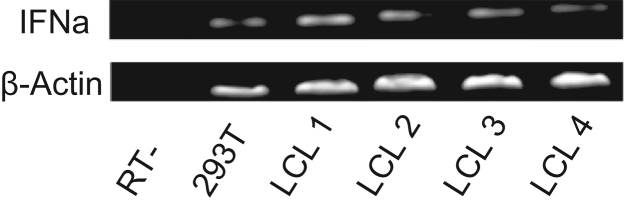
**EBV-transformed lymphoblastoid B cell lines (LCLs) express IFNa.** RT-PCR analysis of IFNa expression in 4 distinct EBV-transformed lymphoblastoid lines was measured in comparison to the positive control cell line, 293T. A no template (NT) negative control is also shown. The housekeeping gene, β-actin, was amplified to demonstrate relative levels of IFNa RNA in each cell line.

One database link, three tables, and one figure are provided in this article. Methyl-seq data from SLE PBMCs segregated based on high or normal numbers of ARID3a^+^ B cells was deposited in NCBI׳s GEO database under the following accession number GEO: GSE84965 [2]. Table 1, Table 2 show qRT-PCR data obtained via Biomark HD for Type I IFN pathway genes from RNA derived from SLE B cells subdivided based on ARID3a levels [1], and for healthy control B cells with or without CpG induced ARID3a expression [4]. IFN signature genes are in bold. Primers for RT-PCR and qRT-PCR are given in Table 3. Fig. 1 shows the results of RT-PCR of IFNa in four EBV-transformed lymphoblastoid B cell lines [3].

## 2 Experimental design, materials and methods

### 2.1 Peripheral blood cells and cell lines

Total peripheral blood mononuclear cells (PBMCs) were obtained via Ficoll purification, and were stained for the pan-B cell marker CD20 and intracellular ARID3a prior to analyses by flow cytometry, as previously described [1]. These data allowed subdivision of SLE samples into ARID3a high and ARID3a normal patient samples, such that ARID3a^H^ SLE samples had numbers of ARID3a^+^ B cells >2 standard deviations above the average numbers of ARID3a^+^ B cells in healthy controls (>9830 ARID3a^+^ B cells/ml), versus ARID3a^N^ (<9830 ARID3a^+^ B cells/ml), as defined previously [1]. B lymphocytes purified by flow cytometric sorting (>97% purity via post-sort analyses) were used immediately for RNA preparation in the case of SLE samples, or in the case of healthy control cells, were grown in complete RPMI media (RPMI 1640, 5×10^−5^ M β-mercaptoethanol, 100 U/ml penicillin, 100 µg*/*ml streptomycin, 2 mM glutamine and 1 mM sodium pyruvate) supplemented with 4% heat inactivated fetal bovine serum (FBS), with or without 5 µg/ml Class CpG oligonucleotide for 24 h, as previously described [4]. Epstein-Barr Virus (EBV)-transformed lymphoblastoid B cell lines (LCLs) were generated from 4 SLE patient samples and maintained in complete RPMI media.

### 2.2 Methyl-seq

To determine if increased expression of ARID3a within SLE patient samples was associated with alterations in DNA methylation, genomic DNA was isolated using standard phenol/Chloroform extraction protocols from total PBMCs obtained from each of two SLE patient samples characterized as ARID3a^H^ and two independent SLE samples characterized as ARID3a low. DNA was fragmented on a Covaris S2 sonicator (Covaris, Woburn, MA) to an average size of ~350 bp in length and methylated DNA was isolated using the MethylMiner Methylated DNA Enrichment Kit (Life Technologies, Carlsbad, CA). Illumina sequencing libraries were prepared from each sample using the Illumina Truseq DNA LT Sample Prep Kit (Illumina, San Diego, CA) by the Genomics Core facility at Oklahoma Medical Research Foundation. Libraries were sequenced on an Illumina Hiseq 2000 instrument with paired-end 100 bp reads. Quality control metrics were assessed with Picard tools v. (https://broadinstitute.github.io/picard/). After sequencing, reads were aligned to the human reference genome hg19 using the aligner BWA-MEM [5] followed by local realignment around problematic indel sequences using the Genome Analysis Tool Kit (GATK) [6]. Genes with statistically significant methylation differences were defined using EpiCenter v. 1-6-1-8 [7]. Methylation differences were tested over promoters of the genes, defined as 2000 bp regions upstream of gene’ transcription start sites. The differentially methylated regions were visualized in the IGV integrative genomics viewer [8]. For visualization in the UCSC Genome Browser BigWig files were created from the final BAM files using a combination of BEDTools [9] and UCSC conversion utilities [10].

### 2.3 Biomark HD assays

Peripheral blood mononuclear cells were isolated from peripheral blood of 6 SLE patients and 2 healthy individuals, and were analyzed for ARID3a expression as described above by flow cytometry. B lymphocytes were enriched from the remaining PBMCs via negative selection using magnetic beads containing other lineage markers, and the remaining cells were stained with CD20 for fluorescence activated cell sorting (FACS) using a FACSAria II (BD Biosciences). Post-sort analyses revealed >98% CD20^+^ B lymphocytes. RNA was isolated, quantified and assessed for integrity using Agilent Total RNA Pico chips on the 2100 Bioanalyzer (Agilent Technologies, Boblingen, Germany). The DELTAgene assay designer was used for primer design for optimal performance on the Biomark HD system. Primer specificity was determined via melting curve analysis at 400 nM. cDNA preparation (Fluidigm preamp master mix, PM100-5580), amplification (Fluidigm, DELTAgene assay kit), qRT-PCR and analyses were all performed as previously described [11]. Data in Table 1, Table 2 are normalized to the housekeeping gene *Hprt1*. A list of primers for the genes assessed is given in Table 3.

### 2.4 IFNa analyses of EBV lines

For qRT-PCR, RNA was extracted using Tri-Reagent (MRC, Inc.) and chloroform:isoamyl alcohol 24:1 (Sigma), precipitated in isopropanol, and collected via centrifugation. cDNA was synthesized at 37 °C for 1 h with M-MLV reverse transcriptase (Promega) and random primers (Promega), and amplified for 40 cycles at 60 °C for 30 s, 72 °C for 1 min, and 95 °C for 30 s for IFNa (IFNA2, IFNA5, IFNA6, IFNA8, IFNA14, IFNA16) gene expression. Amplified products were electrophoresed through 2% agarose gel.

## Figures and Tables

**Table 1:** Upregulated genes in ARID3a^H^ versus ARID3a^N^ SLE B cells.

**Gene**	**ARID3a**^**H**^	**ARID3a**^**N**^	***P-*value**
*ARID3a*	0.6843	0.0692	0.0008
*BCL2*	4.6000	1.3283	0.0081
*BCL2L1*	7.6980	0.3608	0.0049
***EPSTI1***	1958.1900	17.6625	0.0034
***HERC5***	17.4250	1.9833	0.0043
***IFI6***	46.0745	3.7483	0.0227
***IFI27***	1958.1900	17.6625	0.0034
***IFI44***	23.6242	16.6467	0.3387
***IFI44L***	130.6440	29.8092	0.0369
***IFIT3***	33.2118	14.8208	0.0691
***IFNA2***	49.0570	22.9925	0.2194
***IFNAR1***	1.0425	1.2433	0.5319
***IFNB1***	9.3942	1.1575	0.0004
*IRF3*	2.1863	0.1408	0.0008
*IRF5*	1.3742	0.3783	0.0035
*IRF7*	3.5360	0.2510	0.0006
***ISIG15***	5.6233	0.6150	0.0118
***Ly6E***	21.4388	1.5683	0.0023
***MX1***	33.6467	4.3542	0.0009
***MYD88***	4.1236	2.2740	0.0999
***OAS1***	11.7725	0.3267	0.0007
***OAS2***	0.8475	0.2542	0.0847
***OAS3***	6.0257	0.2313	0.0018
***PLSCR1***	17.5975	0.8400	0.0526
***SIGLEC1***	144.2100	55.8713	0.0570
***STAT1***	2.2225	1.0158	0.0660
*TLR 7*	5.7500	2.0808	0.0165
***TLR9***	3.8250	2.8425	0.4706
***USP18***	17.5975	0.8400	0.0010

**Table 2:** Upregulated or downregulated genes in CpG-stimulated versus unstimulated healthy control B cells.

**Gene**	**CpG**	**Unstimulated**	***P*-value**
Upregulated			
***EPST1***	3.14125	1.01875	<0.000001
***HERC5***	6.9225	0.9875	<0.000001
***IFI6***	1.95	1.00125	0.001894
***IFI27***	4.9	1.58	0.001146
***IFI44***	1.805	1.05125	0.003712
***IFI44L***	2.2325	1.23	0.001263
***IFIT3***	3.06375	1.19625	0.000121
*IFNA2*	3.16625	1.07125	0.015070
*IFNAR1*	2.12	1.0075	<0.000001
*IFNB1*	3.3125	1.325	0.017893
***IRF3***	1.94875	1.01875	<0.000001
***IRF5***	1.01375	1.04125	0.710859
***IRF7***	1.5325	0.98625	<0.000001
***ISG15***	0.7825	0.8975	0.385249
***Ly6E***	1.6825	1.015	0.000261
***MX1***	2.61125	1.0375	0.000002
***MYD88***	1.31125	0.96625	0.000083
***OAS1***	4.3175	0.945	<0.000001
***OAS2***	1.7975	0.99	0.000954
***OAS3***	2.1375	1.0725	0.001380
***PLSCR1***	1.5125	0.99625	0.000332
***STAT1***	1.0025	1.01375	0.808948
*TLR7*	5.30625	1.0375	0.000002
*TLR9*	2.28375	0.99875	0.001518

Downregulated			
*BCL2L1*	0.7475	0.9525	0.009423

IFN signature genes are in bold.

**Table 3:** Primer sequences.

**Gene**	**Primer sequence (5′ to 3′)**	**Figure**
***IFIT1***	CTCCTTGGGTTCGTCTATAAATTG AGTCAGCAGCCAGTCTCAG	Fig. 1a in [4]
***HPRT1***	TTGGTCAGGCAGTATAATCC GGGCATATCCTACAACAAAC	Fig. 1a in [4]
***GAPDH***	GCCGCATCTTCTTTTGCGT GCCCAATACGACCAAATCCGT	Fig. 1a in [4]
***CMYC***	ACTCTGAGGAGGAACAAGAA TGGAGACGTGGCACCTCTT	Fig. 1e in [4]
***ARID3A***	AACAAGAAGCTGTGGCGTGA TCATGTATTGGGTCCGCAGG	Fig. 1c in [4]
***ACTIN***	ATCTGGCACCACACCTTCTACAATGAGCTGCG CGTCATACTCCTGCTTGCTGATCCACATCTGC	Fig. 1
***IFNA***	CCTGGCACAAATGAGGAGAA AGCTGCTGGTAAAGTTCAGTATAG	Fig. 2a in [4] Fig. 1
***OAS1***	TACCCTGTGTGTGTGTCCAA AGAGGACTGAGGAAGACAACC	Fig. 3a, 4d in [4]Table 1
***OAS2***	TGGTGAACACCATCTGTGAC CCATCGGAGTTGCCTCTTAA	
***OAS3***	AGGACTGGATGGATGTTAGCC ACTTGTGGCTTGGGTTTGAC	Table 1
***ISG15***	CTGAGAGGCAGCGAACTCA GCTCAGGGACACCTGGAA	Table 1
***PLSCR1***	GTTGTCCCTGCTGCCTTCA TGGGTGCCAAGTCTGAATAACA	Table 2
***HERC5***	TTCAGATCACATGTGGAGATTACC GTTCTGTCCCCAGGCAAAA	Table 1, Table 2
***IFI44***	GGCTTTGGTGGGCACTAATA TGCCATCTTTCCCGTCTCTA	
***IFIT3***	ACTGGCAATTGCGATGTACC GCTCAATGGCCTGCTTCAAA	Table 2
***LY6E***	TGCTCCGACCAGGACAACTA GGCTGTGGCCAAATGTCAC	Table 1, Table 2
***MX1***	ATGCTACTGTGGCCCAGAAA GGCGCACCTTCTCCTCATA	Table 1, Table 2
***USP18***	TGAATGTGGACTTCACCAGGATA GCAGCAGAAGCATCTGGAAA	Table 1
***IFI44L***	GCAAAAGTGAAGCAAGTTCACA GAACCTCACTGCAATCATCCA	Table 1, Table 2
***IFI6***	TGCTACCTGCTGCTCTTCA TCAGGGCCTTCCAGAACC	Table 1
***SIGLEC1***	AGGAGGCGTGTTTGTAAGCA TGTGGCTGCATCAGGATCAA	Fig. 3a in [4]
***IFI27***	TTGTGGCTACTCTGCAGTCA CCCAGGATGAACTTGGTCAA	Table 1
**EPSTI1**	GCAAGAGCAAGAAAGAGCCAAA CCTTGGAGTCGGTCCAGAAAA	Table 1, Table 2
**IRF3**	ACCAATGGTGGAGGCAGTAC TGGGGCCAACACCATGTTA	Fig. 3b, 4e in [4]
**IRF5**	AGATCTACGAGGTCTGCTCCAA CCTCTCCTGCACCAAAAGAGTA	
**IRF7**	GGCAGAGCCGTACCTGTCA ACCGTGCGGCCCTTGTA	
**TLR7**	TCTTCAACCAGACCTCTACATTCC AGCCCCAAGGAGTTTGGAAA	Table 1, Table 2
**TLR9**	TGCAACTGGCTGTTCCTGAA ACAAGGAAAGGCTGGTGACA	Table 2
**MYD88**	CTGCAGAGCAAGGAATGTGAC TGCTGGGGAACTCTTTCTTCA	
**IFNAR1**	AGTGACGCTGTATGTGAGAAAA ACGGGAGAGCAAATAATGCA	Fig. 3b in [4] Table 2
**STAT1**	ATGCTGGCACCAGAACGAA GCTGGCACAATTGGGTTTCAA	
**IFNA2**	AGGATTCAGCGGGAACACAA CAATCTCAAACTCTGGTGGTTCAAA	Table 2
**IFNB1**	ATGAGCAGTCTGCACCTGAA GACTGTACTCCTTGGCCTTCA	Table 2
